# Anti–PD-L1 and anti-CD73 combination therapy promotes T cell response to EGFR-mutated NSCLC

**DOI:** 10.1172/jci.insight.142843

**Published:** 2022-02-08

**Authors:** Eric Tu, Kelly McGlinchey, Jixin Wang, Philip Martin, Steven L.K. Ching, Nicolas Floc’h, James Kurasawa, Jacqueline H. Starrett, Yelena Lazdun, Leslie Wetzel, Barrett Nuttall, Felicia S.L. Ng, Karen T. Coffman, Paul D. Smith, Katerina Politi, Zachary A. Cooper, Katie Streicher

**Affiliations:** 1 Translational Medicine and; 2 Research Early Oncology, AstraZeneca, Gaithersburg, Maryland, USA.; 3 Oncology R&D, Bioscience, AstraZeneca, Cambridge, United Kingdom.; 4 Biologics Engineering, AstraZeneca, Gaithersburg, Maryland, USA.; 5 Department of Pathology, Yale School of Medicine, New Haven, Connecticut, USA.; 6 Alliance Management, AstraZeneca, Gaithersburg, Maryland, USA.; 7 Department of Pathology and Medicine, Yale School of Medicine and Yale Cancer Center, New Haven, Connecticut, USA.

**Keywords:** Immunology, Oncology, Immunotherapy

## Abstract

Treatment with anti–PD-1 and anti-PD-L1 therapies has shown durable clinical benefit in non–small cell lung cancer (NSCLC). However, patients with NSCLC with epidermal growth factor receptor (EGFR) mutations do not respond as well to treatment as patients without an EGFR mutation. We show that EGFR-mutated NSCLC expressed higher levels of CD73 compared with EGFR WT tumors and that CD73 expression was regulated by EGFR signaling. EGFR-mutated cell lines were significantly more resistant to T cell killing compared with WT cell lines through suppression of T cell proliferation and function. In a xenograft mouse model of EGFR-mutated NSCLC, neither anti–PD-L1 nor anti-CD73 antibody alone inhibited tumor growth compared with the isotype control. In contrast, the combination of both antibodies significantly inhibited tumor growth, increased the number of tumor-infiltrating CD8^+^ T cells, and enhanced IFN-γ and TNF-α production of these T cells. Consistently, there were increases in gene expression that corresponded to inflammation and T cell function in tumors treated with the combination of anti–PD-L1 and anti-CD73. Together, these results further support the combination of anti-CD73 and anti–PD-L1 therapies in treating EGFR-mutated NSCLC, while suggesting that increased T cell activity may play a role in response to therapy.

## Introduction

Checkpoint inhibitors that block the PD-1/PD-L1 pathway have revolutionized the clinical care of patients with locally advanced or metastatic non–small cell lung cancer (NSCLC). PD-1/PD-L1 therapeutic antibodies prolonged overall survival of patients in clinical trials and have been approved as first- or second-line treatment of NSCLC (1–5). Although these antibodies can induce durable and potent antitumor immune responses, a significant proportion of patients with NSCLC do not respond to anti–PD-1/PD-L1 treatment.

Activating mutations in epidermal growth factor receptor (EGFR) gene occur in 14% of European patients and 38% of Chinese patients with NSCLC (6). In preclinical studies, anti–PD-1 antibody improved the survival of mice with EGFR-driven lung cancer by enhancing T cell function (7). However, pembrolizumab, nivolumab, and atezolizumab failed to provide survival benefits to patients with EGFR-mutated NSCLC compared with docetaxel in their respective clinical trials (1, 2, 5). A meta-analysis of 3 clinical trials (Checkmate 057, Keynote 010, and POPLAR) has also indicated that patients with NSCLC with EGFR mutations derive less benefits from anti–PD-1/PD-L1 treatment compared with patients without the mutation (8). However, the underlying mechanism that promotes resistance to anti–PD-1/PD-L1 agents in EGFR-mutated NSCLC is still unclear.

We and others have previously demonstrated reduced IFN-γ gene signature and T cell infiltration in EGFR-mutated NSCLC (9, 10), which suggests decreased immunogenicity or suppression of immune response in the tumor microenvironment (TME). Given that PD-L1 expression in tumor tissue is an important biomarker that predicts clinical outcomes to anti–PD-1/PD-L1 treatment (11), it is possible that tumor tissue in EGFR-mutated NSCLC expresses low levels of PD-L1. Pool analysis of 15 published studies and data from The Cancer Genome Atlas show that patients with NSCLC with EGFR mutations have lower PD-L1 expression in their tumor tissue (10). However, other studies have demonstrated an upregulation of PD-L1 in NSCLC with activating EGFR mutations (7, 12). Because reports on PD-L1 status in EGFR-mutated NSCLCs are conflicting, there may be other mechanisms at play that contribute to immunosuppression in the tumors of this patient cohort.

Ecto-5′-nucleotidase (CD73, also known as *NT5E*) is a cell surface ectoenzyme, which is expressed in multiple cell types, including tumor cells, stromal cells, and immune cells. It has been shown that CD73 expressed by tumor cells and Tregs dampens antitumor immunity through catalyzing the conversion of AMP to adenosine (13, 14). Extracellular adenosine impairs the function of immune cells, such as T cells, NK cells, macrophages and DCs. It also promotes the generation of Tregs and myeloid cell–derived suppressor cells, as well as enhancing their suppressive capacity, which further enforces the immunosuppressive milieu in tumor (15). Here, we show that EGFR-mutated NSCLCs expressed higher levels of CD73 compared with those without the mutations. Accordingly, the combination of anti-CD73 and anti–PD-L1 suppressed the growth of EGFR-mutated NSCLC in a xenograft mouse model by enhancing T cell response. Together, these results reveal an EGFR/CD73 axis that suppresses antitumor immunity in EGFR-mutated NSCLC.

## Results

### EGFR signaling promotes CD73 expression in EGFR-mutated NSCLC.

**Figure 1:**
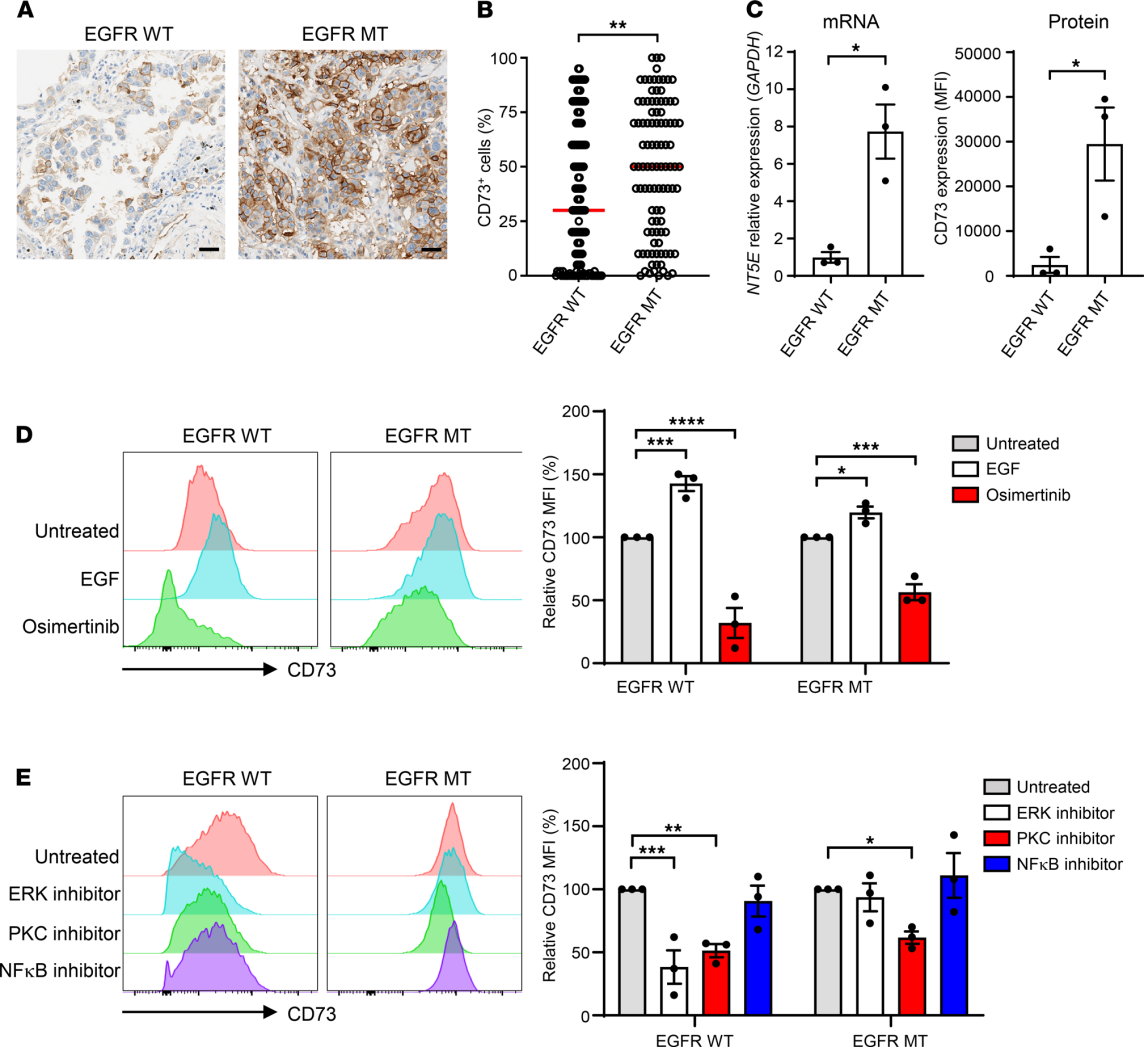
High CD73 expression in EGFR-mutated NSCLC. (**A**) Representative histology images of CD73 expression in EGFR WT and EGFR-mutated (MT) tumors. Scale bar: 50 μm. (**B**) Semiquantitative pathological assessment of CD73 expression in EGFR WT and MT tumor specimens. Each circle represents the data from 1 NSCLC surgical resection. (**C**) CD73 (also known as NT5E) mRNA and protein expression in EGFR WT and MT cell lines. (**D**) CD73 protein expression in EGFR WT and MT cell lines treated with EGF or osimertinib for 48 hours. (**E**) CD73 protein expression in EGFR WT and MT cell lines treated with ERK, PKC, or NF-κB inhibitor for 48 hours. (**C–E**) Data are representative of 2 independent experiments. EGFR WT cell lines were H1355, H157, and H322; EGFR MT cell lines were H1975, HCC2935, and PC9. Student’s *t* test (**B** and **C**) and ANOVA (**D** and **E**) were used. Bars indicate the median in **B** and the mean in **C–E**. Data are shown as the mean ± SEM. *P < 0.05, **P < 0.01, ***P < 0.001, ****P < 0.0001.

We sampled 132 EGFR WT and 99 EGFR-mutated NSCLC FFPE tumor specimens and found that EGFR mutant cells expressed significantly higher CD73 compared with the WT cells (Figure 1, A and B). This is consistent with findings in NSCLC cell lines, where EGFR mutants (H1975, HCC2935, PC9) expressed higher CD73 than the WT cells (H1355, H157, H322) in mRNA and protein levels (Figure 1C). As CD73 is highly expressed in NSCLC with activating EGFR mutations, we next sought to determine the effect of EGFR signaling on CD73 in NSCLC cell lines. Treatment of both WT and mutant cell lines with exogenous EGF led to an increase in CD73 expression. We further confirmed the effect of EGFR signaling on CD73 expression by using osimertinib, a third-generation EGFR tyrosine kinase inhibitor (16). Osimertinib significantly downregulated CD73 levels in both EGFR WT (H1355, H157, H322) and mutant cell lines (H1975, HCC2935, PC9) (Figure 1D). To further demonstrate EGFR signaling–mediated CD73 expression in EGFR-mutated NSCLC, we treated mice bearing PC9 tumors and EGFR^L858R/T790M^ genetically engineered mice (GEM) with osimertinib. In both the PC9 xenograft and GEM model, tumors from osimertinib-treated mice showed a significant reduction of CD73 expression compared with tumors from the control mice (Supplemental Figure 1; supplemental material available online with this article; https://doi.org/10.1172/jci.insight.142843DS1).

We also investigated the underlying molecular mechanisms by which EGFR signal regulates CD73 expression. Cells were treated with chemical inhibitors for ERK, PKC, and NF-κB, which are downstream of the EGFR signaling pathway. A significant reduction of CD73 levels was detected in EGFR WT cells (H1355, H157, H322) when they were treated with inhibitors for either ERK or PKC (Figure 1E). However, only PKC inhibition was able to reduce CD73 in EGFR mutant cells (H1975, HCC2935, PC9); inhibition of ERK was not able to produce this effect (Figure 1E), suggesting a possible involvement of PKC in the regulation of CD73 by EGF signaling in the mutants. Together, these data indicate that EGFR-mutated NSCLC express increased levels of CD73, which is driven by EGFR signaling.

### EGFR-mutated NSCLC suppresses T cell–mediated killing.

Because the immune response may be suppressed in the TME of EGFR-mutated NSCLC, as suggested by previous studies (9, 10), we investigated the sensitivity of EGFR WT and EGFR-mutated cells to T cell–mediated killing in an antigen-specific manner. To this end, we developed an in vitro assay that measured T cell–mediated killing in response to MART-1 peptide using the xCelligence system. MART-1 peptide (Melan-A_26–35_) is a human HLA-A2–restricted T cell epitope derived from melanoma (17). We enriched MART-1–specific T cells in PBMCs by stimulating naive CD8^+^ T cells with MART-1 peptide–pulsed DCs and cytokines. MART-1–enriched T cells (≈50% of which were MART-1 specific; data not shown) were cultured with EGFR WT (H1355) and EGFR-mutated (HCC2935, PC9) NSCLC cell lines that were HLA-A2 positive, in the presence of MART-1 peptide.

**Figure 2:**
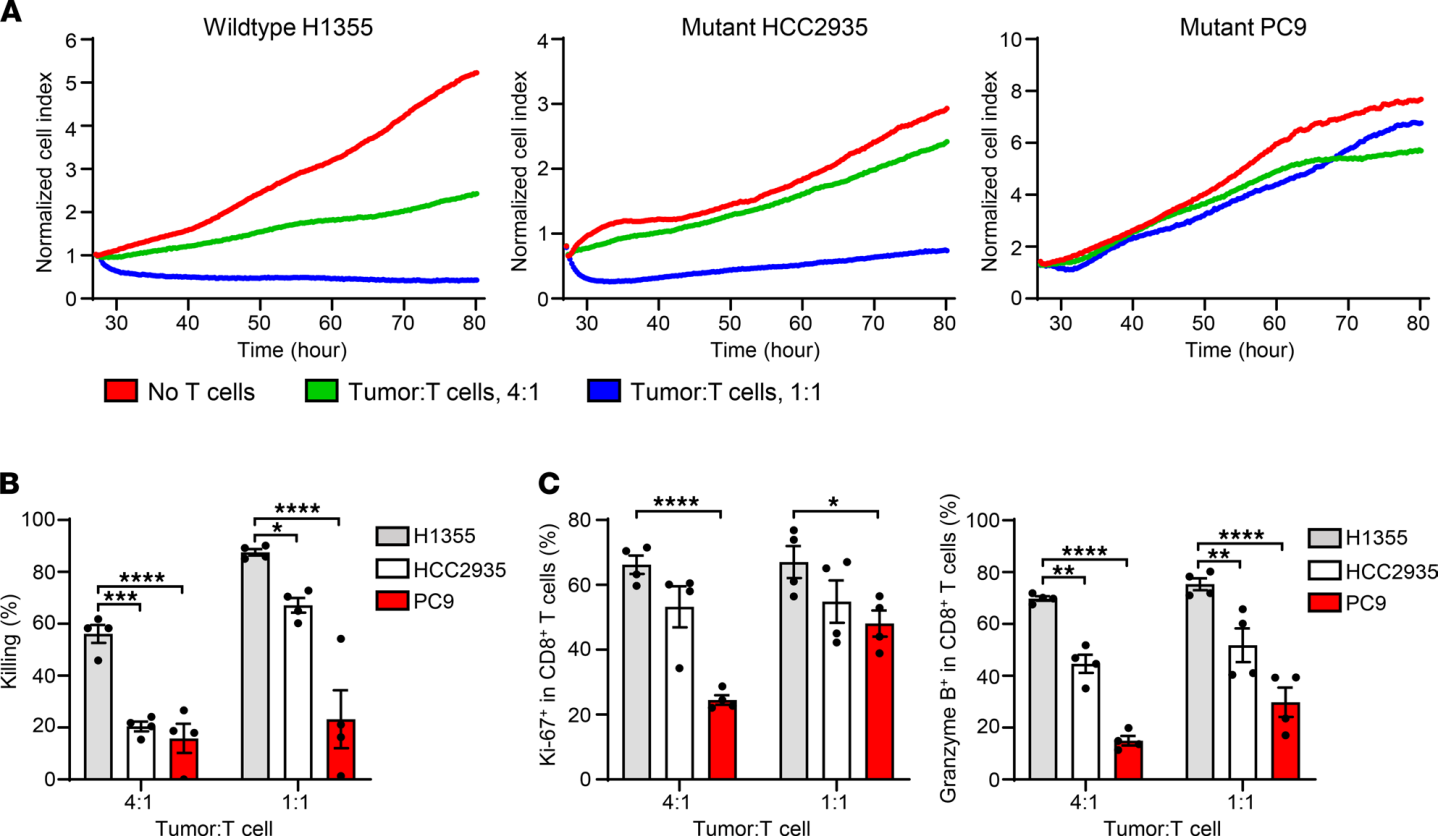
EGFR-mutated NSCLCs are more resistant to T cell killing. MART-1–enriched T cells were cultured with the indicated ratio of H1355, HCC2935, or PC9 cells plus MART-1 peptide for 3 days. (**A**) Representative chart of cell index. (**B**) T cell killing was determined by the changes in cell index caused by T cells compared with tumor cells cultured alone. (**C**) Frequencies of Ki-67^+^ and granzyme B^+^ in CD8^+^ T cells cultured with tumor cells. (**A**) Data are representative of 4 independent experiments or (**B** and **C**) are pooled from 4 independent experiments. ANOVA was used. Bars indicate the mean. Data are shown as the mean ± SEM. *P < 0.05, **P < 0.01, ***P < 0.001, ****P < 0.0001.

In this assay, EGFR WT H1355 cells were found to be more sensitive to T cell–mediated killing compared with the mutant HCC2935 and PC9 cells (Figure 2, A and B). MART-1–enriched T cells effectively killed more than 50% of H1355 cells, even at a high tumor-to–T cell ratio of 4:1, and killing of target cells was further enhanced to around 90% when the number of T cells increased (Figure 2B). In comparison, more than 50% of killing of HCC2935 cells was only achieved at a low tumor-to–T cell ratio of 1:1. MART-1–enriched T cells were not able to mediate substantial killing of PC9 cells, even at a low tumor-to–T cell ratio (Figure 2, A and B). Consistently, T cells that were cultured with the EGFR-mutated cells, especially PC9 cells, showed significantly lower Ki-67 and granzyme B levels compared with T cells that were cultured with H1355 cells (Figure 2C). These results altogether demonstrate that EGFR-mutated NSCLC cells are more resistant to T cell–mediated killing through suppression of T cells.

### Combination of anti-CD73 and anti–PD-L1 promotes T cell response against EGFR-mutated NSCLC.

**Figure 3:**
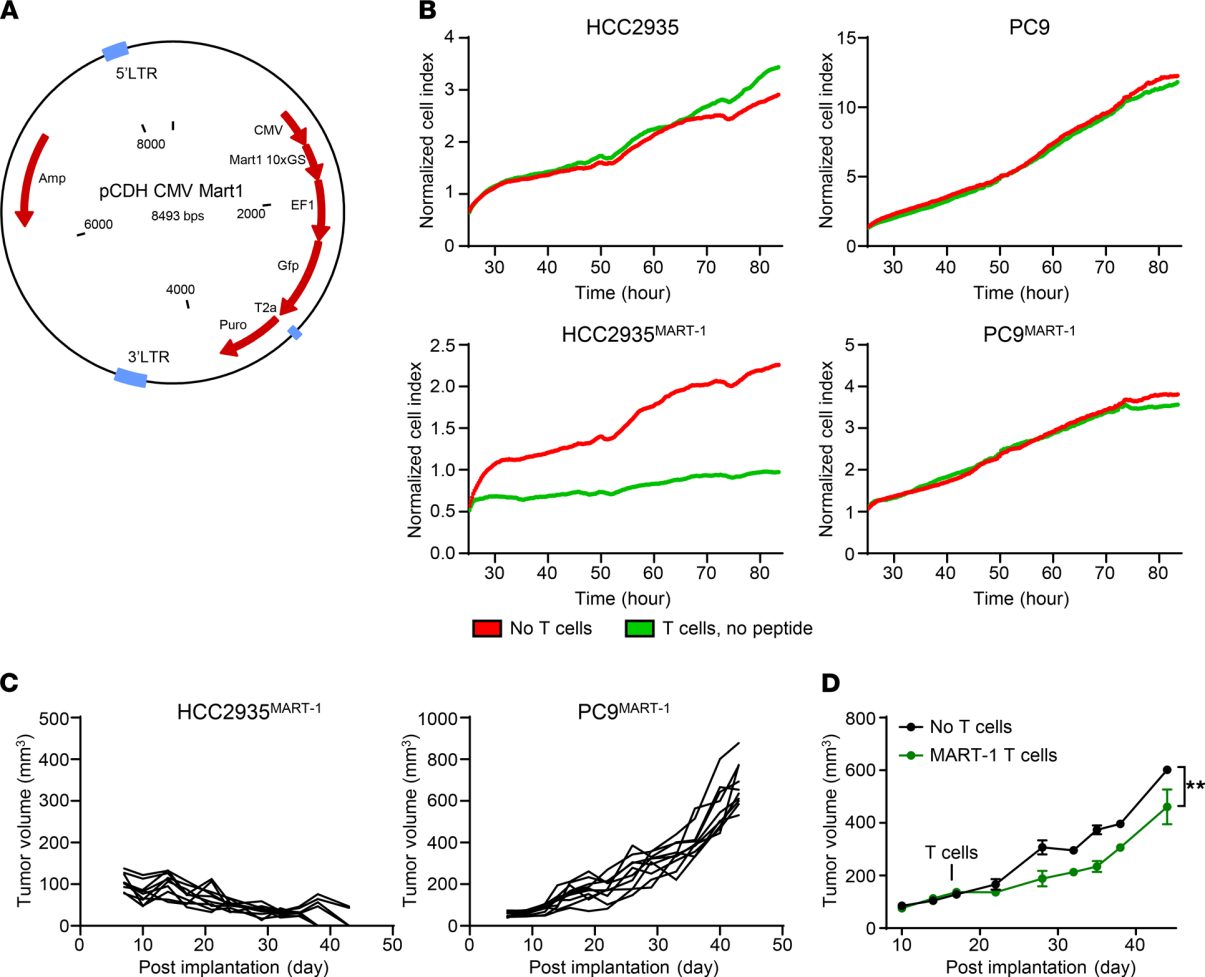
Generation of MART-1–expressing NSCLC cell lines. (**A**) Schematic map of MART-1–expressing plasmid. (**B**) Proliferation index of the indicated cell lines either cultured alone (red) or with MART-1–enriched T cells at a 1:1 ratio in the absence of MART-1 peptide (green). (**C**) Tumor volume of recipient mice after implantation of the indicated cell lines. (**D**) Tumor volume of recipient mice after implantation of PC9^MART-1^ cells with or without MART-1–enriched T cells. Data are representative of 2 independent experiments. ANOVA was used. Data are shown as the mean ± SEM. **P < 0.01.

**Figure 4:**
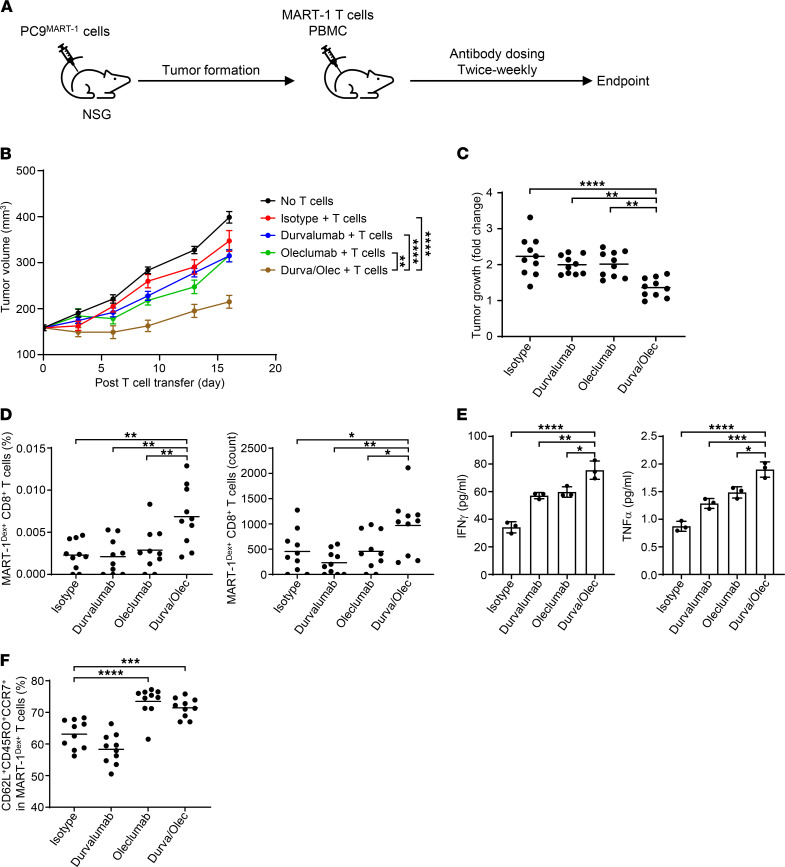
The combination of durvalumab and oleclumab promotes T cell killing of EGFR-mutated NSCLC. (**A**) Experimental workflow of antibody treatment in a mouse model of EGFR-mutated NSCLC. (**B**) Tumor volume of recipient mice after T cell transfer. (**C**) Fold change in tumor volume between the start and end of antibody treatment. (**D**) Frequencies and absolute numbers of MART-1 dextramer–labeled (Dex-labeled) CD8^+^ T cells in the tumor. (**E**) Cytokine production by MART-1 dextramer–labeled CD8^+^ T cells from treated mice, after culture with irradiated T2 cells and MART-1 peptide for 72 hours. (**F**) Frequencies of CD62L^+^CD45RO^+^CCR7^+^ cells in MART-1 dextramer–labeled CD8^+^ T cell population in the spleen. Data are representative of 2 independent experiments independent experiments. In **C**, **D**, and **F**, each circle represents the data from 1 mouse. ANOVA was used. Bars indicate the mean. Data are shown as the mean ± SEM in **B** and mean ± SD in **E**, *P < 0.05, **P < 0.01, ***P < 0.001, ****P < 0.0001.

Next, we developed an EGFR-mutated NSCLC mouse model using genetically modified HCC2935 and PC9 cells that overexpressed MART-1 peptide (Figure 3A). MART-1–enriched T cells effectively killed MART-1–overexpressing HCC2935 (HCC2935^MART-1^) cells in vitro, even in the absence of exogenous MART-1 peptide. Similar to regular PC9 cells, MART-1–overexpressing PC9 (PC9^MART-1^) cells were not sensitive to T cell–mediated killing (Figure 3B). When implanted into NSG mice, only PC9^MART-1^ cells, but not HCC2935^MART-1^ cells, were able to develop into tumors (Figure 3C). MART-1–enriched T cells and PBMCs together reduced tumor growth in this mouse model but were unable to eradicate tumors (Figure 3D). We examined if T cell–mediated killing of EGFR-mutated NSCLC can be enhanced in vivo by targeting either CD73 and PD-L1 alone or in combination. MART-1–enriched T cells and PBMCs were transferred into NSG mice after PC9^MART-1^ cells had formed tumors (≈160 mm^3^). Mice were then treated with an isotype control antibody, anti–PD-L1 (MEDI4736, durvalumab) (4), anti-CD73 (MEDI9447, oleclumab), or a combination of anti–PD-L1 and anti-CD73 (durvalumab/oleclumab) (Figure 4A). Oleclumab is a human monoclonal antibody that specifically binds to and inhibits the ectonucleotidase activity of CD73 (18). Although durvalumab or oleclumab alone did not induce tumor growth inhibition (TGI) compared with isotype control, mice treated with a combination of durvalumab and oleclumab had significantly smaller tumors compared with mice in other groups (Figure 4B). Furthermore, tumor growth was much lower in mice treated with the antibody combination when tumor volumes at the start and end of treatment were compared (Figure 4C).

We also identified MART-1–specific CD8^+^ T cells within tumor-infiltrating cells using MART-1 dextramer. Consistent with enhanced TGI, the durvalumab/oleclumab combination significantly increased the frequency and number of MART-1–specific (MART-1^Dex+^) CD8^+^ T cells in tumors compared with isotype control or either antibody alone (Figure 4D). In addition to the frequency of MART-1–specific CD8^+^ T cells, we investigated the effect of the antibody combination on effector T cell function. MART-1 dextramer–labeled CD8^+^ T cells were magnetically sorted from mice in the above-mentioned in vivo experiment, and the cells were restimulated with MART-1 peptide in vitro. MART-1–specific CD8^+^ T cells from mice treated with durvalumab and oleclumab produced much higher levels of IFN-γ and TNF-α upon peptide stimulation compared with T cells in other groups of mice (Figure 4E), suggesting an enhancement of T cell function. Interestingly, targeting of CD73 led to an increase in CD62L^+^CD45RO^+^CCR7^+^ central memory T (Tcm) cells in the spleen. This was seen when oleclumab was given to mice either alone or in combination with durvalumab (Figure 4F). However, the antibody treatments did not affect the memory subsets of tumor-infiltrating MART-1–specific CD8^+^ T cells (data not shown). Overall, these data indicate that the durvalumab/oleclumab combination suppresses the growth of EGFR-mutated NSCLC by promoting T cell response.

### Combination of durvalumab and oleclumab enhances expression of immune-related genes and signatures.

**Figure 5:**
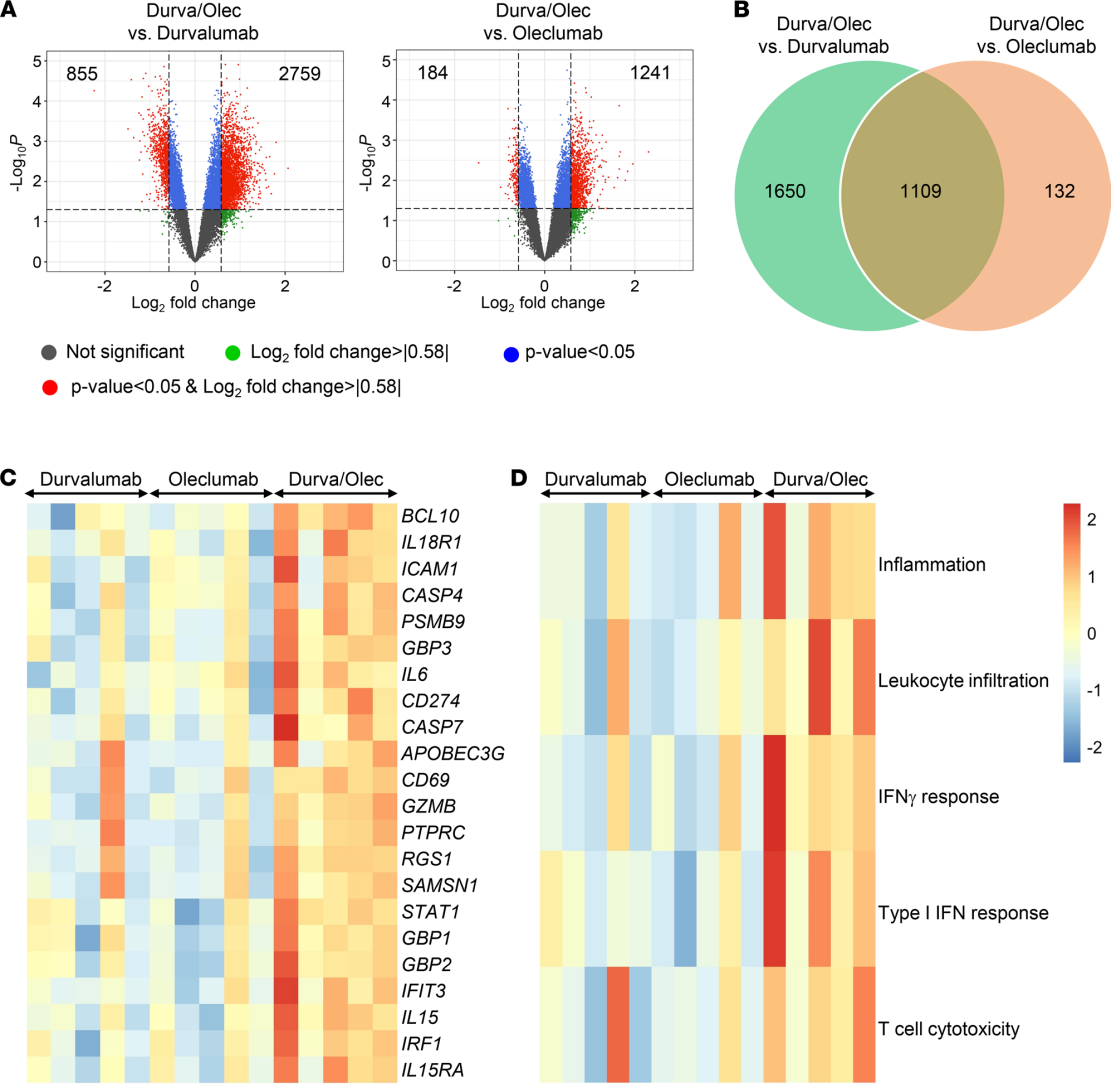
Enhanced immune signatures in durvalumab/oleclumab–treated tumors. (**A**) Volcano plots of comparative analysis of gene expression in durvalumab/oleclumab–treated tumor with respect to durvalumab- or oleclumab-treated tumors. Genes with significant interaction (*P* < 0.05, fold change > 1.5 or < –1.5) are shown in red. (**B**) Venn diagram illustration of shared and unique genes upregulated (*P* < 0.05, fold change > 1.5) in durvalumab/oleclumab–treated tumor with respect to durvalumab- or oleclumab-treated tumors. (**C** and **D**) Heatmaps of selected (**C**) genes and (**D**) gene signatures upregulated or downregulated in tumors from durvalumab-, oleclumab-, and durvalumab/oleclumab–treated mice. Signature scores based on the median of gene signature components were calculated. A color gradient ranging from blue to red indicates the scores of induced (red) or repressed (blue) genes and signatures. Moderated *t* test was used. Each box represents the data from 1 mouse.

To better understand why the durvalumab/oleclumab combination induced better therapeutic effects than either antibody alone, we carried out comparative analysis of gene expression in the tumor tissue. The durvalumab/oleclumab combination differentially upregulated 2759 and 1241 genes in the treated tumors when compared with durvalumab and oleclumab alone, respectively (fold change ≥ 1.5 and *P* < 0.05) (Figure 5A). Among the top 10 most differentially expressed genes between combination and monotherapies, there are genes that are involved in infiltration, tissue retention, and adhesion of T cells, such as *RGS1* and *PSMB9* (Table 1) (19–23). When analyzing the overlap of 1109 upregulated genes between the 2 comparisons (Figure 5B), we found genes that were indicative of an increase in inflammatory response and apoptosis as well as genes regulated by IFN-γ (Figure 5C). Consistently, durvalumab/oleclumab combination treatment increased gene signatures associated with T cell–mediated cytotoxicity, inflammation, leukocyte infiltration, IFN-γ response, and type I IFN response (Figure 5D and Supplemental Table 1). Apart from immune-related genes and signatures, we performed gene ontology analysis to report differentially expressed genes that are involved in biological process, molecular function, and cellular component, between combination and monotherapies (Supplemental Figure 2). Taken together, the data suggest that combined targeting of CD73 and PD-L1 is more effective than monotherapies in promoting T cell function and response.

## Discussion

A mounting body of evidences indicate that CD73 is highly expressed in several cancer types and it correlates with poor clinical outcome (24–26). Here, we revealed upregulation of CD73 expression in EGFR-mutated NSCLC, which contributed to suppression of T cell response. This notion is supported by previous findings that CD73 on tumor cells impairs antitumor T cells (27). Furthermore, treatment with a combination of anti–PD-L1 and anti-CD73 enhanced TGI and T cell response against EGFR-mutated NSCLC tumors, compared with treatment with anti–PD-L1 alone. Therefore, our results demonstrate that immunosuppression in TME by CD73 may be the underlying mechanism by which EGFR mutations limit the therapeutic effects of anti-PD1/PD-L1 agents.

We found that CD73 expression in NSCLC is promoted by EGFR signaling, which provides an explanation for high levels of CD73 in NSCLC with activating EGFR mutations. Osimertinib has been shown to selectively inhibit mutated EGFR, with lower activity against WT EGFR (16). However, we showed that osimertinib reduces CD73 expression in both EGFR WT and EGFR-mutated cell lines, indicating the effectiveness of osimertinib in targeting EGFR-regulated CD73 expression. Our data also suggest that EGFR WT and EGFR-mutated cells might be fundamentally different with regards to their EGFR-mediated regulation of CD73 expression. Although both ERK and PKC inhibitors reduced CD73 expression in EGFR WT cells, reduction of CD73 was only detected in EGFR-mutated cells when treated with PKC inhibitor. This result indicates that EGFR-mutated cells upregulate CD73 through EGFR-PKC activation, whereas ERK is not involved in the process. This is in line with several studies that demonstrated the critical role of PKC in NSCLC with EGFR mutation (28–30).

When tumor-bearing mice were treated with durvalumab alone, it did not suppress tumor growth compared with isotype control, which closely mimicked the limited therapeutic effect of anti–PD-1 and anti–PD-L1 antibodies on EGFR-mutated NSCLC. The effects of adenosine in suppressing activation and effector-memory differentiation of CD8 T cells are well demonstrated (31–34), which may explain why targeting of CD73 preferentially expands Tcm cells in our study. Furthermore, recent work has indicated that Tcm cells are more sensitive to the suppressive effects of adenosine due to their higher expression of adenosine receptors, compared with other T cell subsets (35). Consequently, treatment with oleclumab may selectively relieve Tcm cells from the inhibition of adenosine, through blocking the conversion of AMP to adenosine by CD73. Our results are also consistent with those of previous studies that demonstrated central memory T cells and homing to secondary lymphoid tissue confer increased antitumor immunity (36). Nevertheless, superior antitumor activities were only achieved by combination treatment with durvalumab and oleclumab, which suggests that CD73 and PD-L1 jointly suppress T cells in the TME and that targeting of both CD73 and PD-L1 is required to induce optimal T cell response against EGFR-mutated NSCLC. Enhanced antitumor activity induced by durvalumab/oleclumab was most likely due to increases in T cell infiltration and cytokine production. Consequently, increased expression of immune-related genes and signatures was only observed in tumors treated with the antibody combination. A recent study has also demonstrated that the combination of erlotinib and anti–PD-1 enhances antitumor activities and CD8^+^ T cell infiltration in the TME in an EGFR-mutated NSCLC mouse model (37). It may be possible that inhibition of EGFR signaling by erlotinib reduces CD73 expression in the tumors, which then synergizes with anti–PD-1 to increase T cell response. However, high incidence of toxicities have been reported in patients with NSCLC treated with a combination of EGFR inhibitor and PD-1/PD-L1 blockade (38, 39). The combination of anti-CD73 and anti–PD-1/PD-L1 blockade or EGFR inhibitor may be a safer approach for patients, while providing antitumor effects. The combination of oleclumab with durvalumab or osimertinib for treating patients with EGFR-mutated NSCLC is being evaluated in clinical trials. We recently showed that the combinations had a manageable safety profile and that durable responses were observed in some patients (40, 41).

We have demonstrated in this study that resistance to anti–PD-L1 in EGFR-mutated NSCLC could be reversed by targeting CD73 and PD-L1 simultaneously. The antibody combination enhanced TGI, as well as T cell and inflammatory response, compared with either anti-CD73 or anti–PD-L1 alone. However, the antibody combination did not completely eradicate tumors. This may be caused by constraints of our xenograft mouse model. Given that we only transferred 1 dose of MART-1–enriched T cells and PBMCs to the NSG mice, the lack of a constant supply of immune cells for killing the tumor may underrepresent the therapeutic effect of the antibody combination. In addition, the absence of human cytokines and chemokines in the mouse system may not support optimal killing and function of the tumor antigen–specific T cells. Further investigation of this aspect may be warranted in future studies using different models, such as humanized PDX models. Alternatively, it is possible that there are other mechanisms that contribute to immunosuppression in the TME of EGFR-mutated NSCLC. Previous studies have demonstrated that EGFR signaling and extracellular adenosine enhance frequencies and suppressive function of Tregs (42–44). Moreover, EGFR-containing exosomes derived from lung cancer biopsies have been found by others to induce the generation of tumor antigen–specific Tregs, which suppresses antitumor T cells (45). Consistently, EGFR blockade, as well as suppression of CD73 expression, reduces Treg frequencies in EGFR-mutated NSCLC (37, 46). Further studies will be required to determine the association between Tregs and lack of response to anti–PD-1/PD-L1 treatment in EGFR-mutated NSCLC. Despite the limitations of this study, our results provide a critical understanding of the effects of anti–PD-L1 and anti-CD73 combination in the treatment of EGFR-mutated NSCLC, which could have important implications for continued clinical development of this combination therapy.

## Methods

### NSCLC samples and variant calling.

NSCLC FFPE samples were acquired from Manchester Cancer Research Center, TriStar, Asterand, and Nottingham Health Science Biobank. EGFR mutation status was determined by the providers or verified internally by whole-exome sequencing. Sequencing data were analyzed using bcbio-nextgen (https://bcbio-nextgen.readthedocs.io/en/latest/). Reads were aligned to the hg38 reference using bwa v0.7.15, a quality control report was generated using multiqc (https://multiqc.info/), and sequencing duplicates for each UMI were collapsed into a single consensus read using fgbio (http://fulcrumgenomics.github.io/fgbio/). Samples were subsequently excluded from further analysis if deemed to be of low quality (i.e., sequencing depth of coverage, <50×; on-target reads, <50%; tumor purity, ≤20%). Variant calling was performed using VarDict v1.5.4 (47) down to a variant allele frequency of 1% (before filtering and curation), and variant effects were annotated by snpEff. Filtering of common polymorphisms was performed as per VarDict best practices. In addition, variants were removed if they satisfiy any one of the following criteria: (a) variant allele frequency, >95%; (b) variant allele frequency, <5%; (c) <4 alternative reads supporting the variant; and (d) variant depth, <50. Potential FFPE artifacts were annotated using the DKFZ bias filter (https://github.com/DKFZ-ODCF/DKFZBiasFilter [branch name: master; commit ID: a486939]), and variants marked as “damage” were removed. EGFR variants of unknown significance were removed before determining sample EGFR mutation status.

### Immunohistochemistry.

CD73 immunohistochemistry was performed on the Ventana Discovery platform using anti-CD73 antibody (Abcam, ab124725). CD73 expression on tumor cells was scored semiquantitatively by a pathologist blinded to EGFR status. The total percentage of tumor cells with apical/luminal or complete circumferential membrane staining was estimated.

### Cell lines.

Cell lines H1355, H1666, H157, H322, H1975, HCC2935, and T2 were obtained from ATCC. PC9 cells were obtained from the European Collection of Authenticated Cell Cultures. All cell lines were cultured in RPMI medium with GlutaMAX, penicillin, streptomycin, and 10% fetal bovine serum. MART-1–expressing HCC2935 and PC9 cells were generated via lentiviral transduction. Briefly, MART-1 expression plasmid was synthesized by GeneArt (Thermo Fisher Scientific) and subcloned into pCDH1-CMV-MCS-EF1-GFP-T2a-puro lentiviral vector (System Biosciences). 293X cells (internal cell bank) were transfected with pPACKH1 and lentiviral vector using 293Fectin (Gibco). Lentiviral supernatant was harvested and concentrated 48 hours after transfection. PC9 and HCC2935 cells were transduced through centrifugation with concentrated lentivirus and polybrene (MilliporeSigma). The transduced cells were cultured in medium supplemented with puromycin (Gibco) for at least 1 week to establish stable cell lines.

### Real-time PCR.

Total RNA was derived from cells using the RNeasy Mini Kit (Qiagen) and reversed transcribed using the High Capacity cDNA Reverse Transcription Kit (Applied Biosystems). Quantitative real-time PCR was performed according to the TaqMan Gene Expression Master Mix (Applied Biosystems) protocol with the following TaqMan primers: *GAPDH*, Hs02758991_m1; and *NT5E*, Hs00159686_m1.

### Flow cytometry analysis.

The following antibodies were used for the analysis of surface and intracellular markers: anti-CD73 (2C5) antibody was discovered at AstraZeneca (18) and conjugated to fluorochrome in house; anti-CD73 (TY/11.8) was purchased from BioLegend; anti-Ki-67 (SolA15), anti-granzyme B (GB11), and anti-CD8 (OKT8) were purchased from eBioscience; and FcR blocking reagent was purchased from Miltenyi Biotec. Intranuclear staining was performed using Fixation/Permeabilization buffer solution (eBioscience) according to the manufacturer’s instructions. MART-1 dextramer was purchased from Immudex, and the staining was performed according to the manufacturer’s instructions. In some experiments, cells were treated with EGF (50 ng/ml), EGFR inhibitors (osimertinib, 1 μM, AstraZeneca), NF-κB inhibitor (IKK inhibitor VII, 1 μM, MilliporeSigma), ERK inhibitor (LY3214996, 1 μM, Selleckchem), or PKC inhibitor (Go 6983, 10 μM, MilliporeSigma) for 48 hours before analysis. Stained cells were analyzed on a LSRFortessa (BD Biosciences), and data were analyzed with FlowJo software.

### In vivo treatment with EGFR inhibitor.

PC-9 xenografts were established by subcutaneous implantation of 5 × 10^6^ cells per animal in the dorsal right flank of female SCID mice. All mice were older than 6 weeks of age at the time of cell implant. Mice were randomized at a tumor volume of between 0.2 and 0.4 cm^3^. Randomization for animal studies was based on initial tumor volumes to ensure equal distribution across groups. Mice were then treated orally with either vehicle or osimertinib at 25 mg/kg daily for 3 days. Tumors were excised 24 hours after final dosing. The EGFR^L858R/T790M^ GEM model at Yale University was also used in some experiments. The generation of doxycycline-inducible EGFR^L858R/T790M^ mice has been previously described (48). Mice were treated with 25 mg/kg osimertinib daily, Monday through Friday, for 2 weeks after they had been on doxycycline for approximately 3 months and developed tumors.

### Generation of MART-1–enriched T cells.

DCs were generated by isolating CD14^+^ cells from PBMCs (Discovery Life Sciences Inc.) using the CD14 enrichment kit (Miltenyi) according to the manufacturer’s instructions and cultured in Dendritic Cell media (Cellgro) containing 1% human AB serum supplemented with rhIL-4 (100 ng/ml) and rhGM-CSF (100 ng/ml, Peprotech). The media was removed on day 4 of culture and replaced with fresh media containing 1% human AB serum supplemented with rhIL-4, rhGM-CSF, and hIFN-γ (10 ng/ml, Peprotech), lipopolysaccharide (10 ng/ml, MilliporeSigma), and MART-1 peptide (2.5 μg/ml, Anaspec). On day 5, the mature DCs were harvested and irradiated at 30 Gy. Naive CD8^+^ T cells were isolated using the Naive CD8 Isolation kit (StemCell) and cultured with irradiated DCs in media containing 5% human AB serum supplemented with rhIL-21 (30 ng/ml), MART-1 peptide, rhIL-7 (5 ng/ml), and rhIL-15 (5 ng/ml). Fresh media supplemented with rhIL-7 and rhIL-15 was added on day 3, 5, and 7. Cells were harvested on day 10 and antigen specificity was assessed by staining with MART-1 Dextramer (Immudex) and analyzing by flow cytometry.

### In vitro T cell killing assay.

In vitro T cell killing was analyzed on an xCELLigence RTCA instrument (ACEA Biosciences). Briefly, tumor cells were added into a xCELLigence E-Plate. The plate was loaded into the instrument to begin data acquisition. Twenty-four hours later, MART-1–enriched T cells were added into the plate, at indicated tumor–T cell ratios, together with MART-1 peptide (500 ng/ml). The cells were harvested 3 days later. T cell killing was determined by the percentage of changes in cell index or impedance in electric current caused by T cells compared with tumor cells cultured alone. The changes in cell index were expressed as changes in area under the curve, which was calculated using 
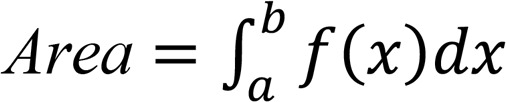
 with Microsoft Excel.

### Antibody treatment in tumor-bearing mice.

NSG mice at 6–8 weeks of age were obtained from The Jackson Laboratories. 5 × 10^6^ MART-1–expressing PC9 cells were subcutaneously injected into the right flank of recipient mice. Tumors were staged and randomized by tumor volume when they reached approximately 140–240 mm^3^. One day before randomization, mice received an injection of 20 mg/kg nonspecific blocking antibodies (hIgG1 and mIgG1, AstraZeneca). A mixture of 5 × 10^6^ MART-1–enriched T cells and 5 × 10^6^ donor-matched PBMCs was injected into mice on the day of randomization. Twenty-four hours after transfer of the cells, 20 mg/kg targeted antibodies (anti-CD73, MEDI9447 oleclumab; anti–PD-L1, MEDI4736 durvalumab; isotype control: NIP228 hIgG1) with 20 mg/kg of blocking isotype antibodies were administered twice per week.

### In vitro peptide challenge.

Cells from tumors and spleens were stained with MART-1 dextramer, followed by anti–PE-labeled microbeads (Miltenyi Biotec) according to the manufacturer’s instructions. The labeled cells were purified by magnetic cell sorting (Miltenyi Biotec) and cultured with irradiated T2 cells (100 Gy) plus MART-1 peptide at 100 ng/ml for 72 hours. IFN-γ and TNF-α in culture supernatant were measured by the V-PLEX Proinflammatory Panel 1 Kit (Meso Scale Discovery) according to the manufacturer’s instructions.

### Gene array analysis.

Total RNA was derived from cells using the RNeasy Mini Kit (Qiagen) and converted to biotin-labeled cRNA using the MessageAmp Premier RNA Amplification Kit (Thermo Fisher Scientific) according to the manufacturer’s instructions. Biotin-labeled cRNA was fragmented and hybridized on Human Genome U133 Plus 2.0 GeneChip arrays (Affymetrix) according to the manufacturer’s instructions. Data capture and array quality assessments were performed with the GeneChip Operating Software tool. Normalization of gene expression profiles was performed by using robust multiarray average method, and the normalized gene expression data were presented as log_2_-transformed values. Probe sets with highest sample variance among multiple probe sets per gene were selected and used for downstream analyses. Differential gene expression analysis was carried out by R package LIMMA with linear modeling and empirical Bayes moderation. The expression of gene signatures, including inflammation (49), leukocyte infiltration (50), IFN-γ response (51), type I IFN response (52), and T cell cytotoxicity (Dr. Jixin Wang, personal communication, AstraZeneca, Gaithersburg, Maryland, USA), was evaluated by the median of gene signature components. The heatmaps of corresponding gene signature expression were drawn with R package pheatmap. Gene ontology enrichment analysis, including biological process, molecular function, and cellular component, was performed using the R package enrichR for upregulated and downregulated genes (53). The array data can be found at NCBI GEO (GSE190731).

### Statistics.

Statistical analysis was performed using unpaired 2-tailed Student’s *t* tests or 2-way ANOVA (Figures 1–3 and Figure 4, A and B) or 1-way ANOVA (Figure 4, C–F) in GraphPad Prism and moderated 1-tailed *t* test in LIMMA.

### Study approval.

Treatment of PC9 tumor-bearing mice with osimertinib was conducted at AstraZeneca (UK), in accordance with UK Home Office legislation, the Animal Scientific Procedures Act 1986, as well as the AstraZeneca Global Bioethics policy, and was approved by AstraZeneca Ethical Review. Treatment of EGFR^L858R/T790M^ GEM with osimertinib was conducted at Yale University in agreement with the NIH *Guide for the Care and Use of Laboratory Animals* (National Academies Press, 2011) and was approved by the Yale University Institutional Animal Care and Use Committee. Treatment of PC9 tumor-bearing mice with antibodies and T cells was conducted at AstraZeneca (USA) in accordance with and with approval of AstraZeneca’s Institutional Animal Care and Use Committee.

## Author contributions

ET designed and performed experiments, analyzed data, and wrote the manuscript. KM, JW, SLKC, NF, JHS, JK, YL, LW, and KTC designed and performed experiments. PM, BN, and FSLN designed and performed experiments and analyzed data for CD73 expression in NSCLC specimens. PDS, KP, and ZAC provided critical scientific input. KS conceived, initiated, and supervised the whole study, designed experiments, and wrote the manuscript.

## Supplementary Material

Supplemental data

## Figures and Tables

**Table 1:**
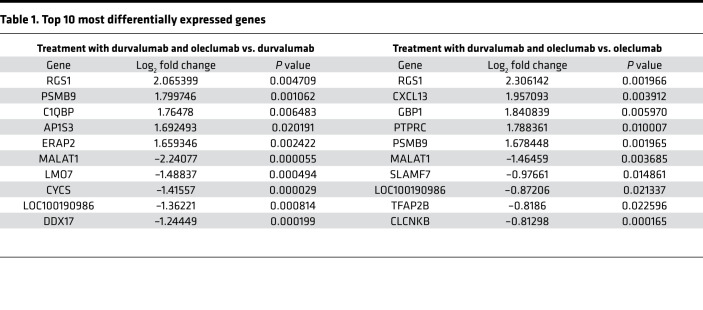
Top 10 most differentially expressed genes

## Version 1 02/08/2022

Electronic publication
