# Supplementary material for: Anti–PD-L1 and anti-CD73 combination therapy promotes T cell response to EGFR-mutated NSCLC
Source: JCI Insight. 2022 Feb 8;7(3):e142843. doi: 10.1172/jci.insight.142843 (PMC8855814; doi:10.1172/jci.insight.142843)
Supplement: Supplemental data [file jciinsight-7-142843-s121.pdf]

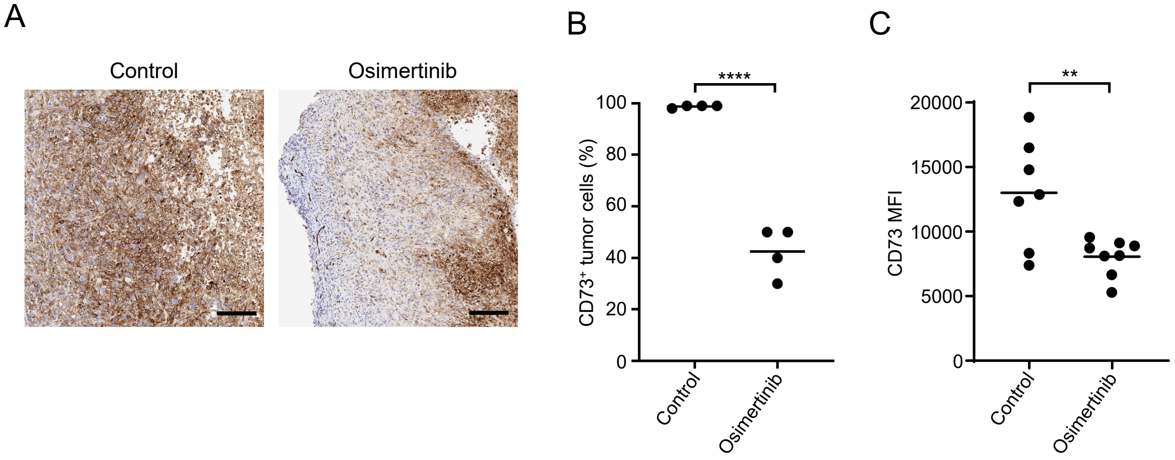

Supplemental Figure 1. EGFR inhibitor reduces CD73 expression in EGFR-mutated tumors *in vivo*
(A) Representative histology images and (B) Semi-quantitative pathological assessment of CD73 expression in PC9 tumors, treated with or without osimertinib. (C) CD73 protein expression in tumors of GEM model, treated with or without osimertinib. Each circle represents the data from one mouse. Scale bar, 200 µm. Student's *t*-test was used; bars (mean), $**P < 0.01$ and $****P < 0.0001$.

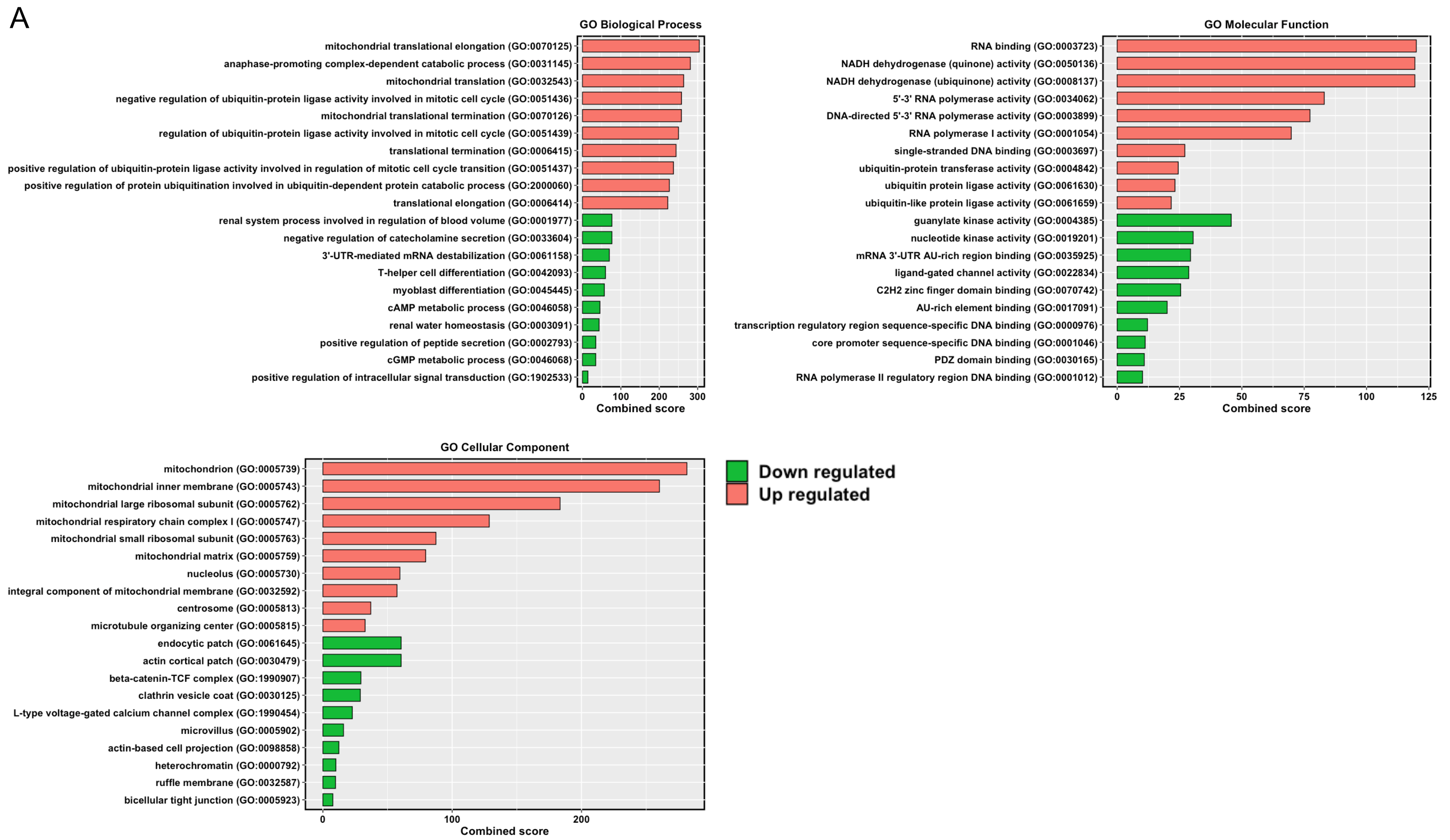

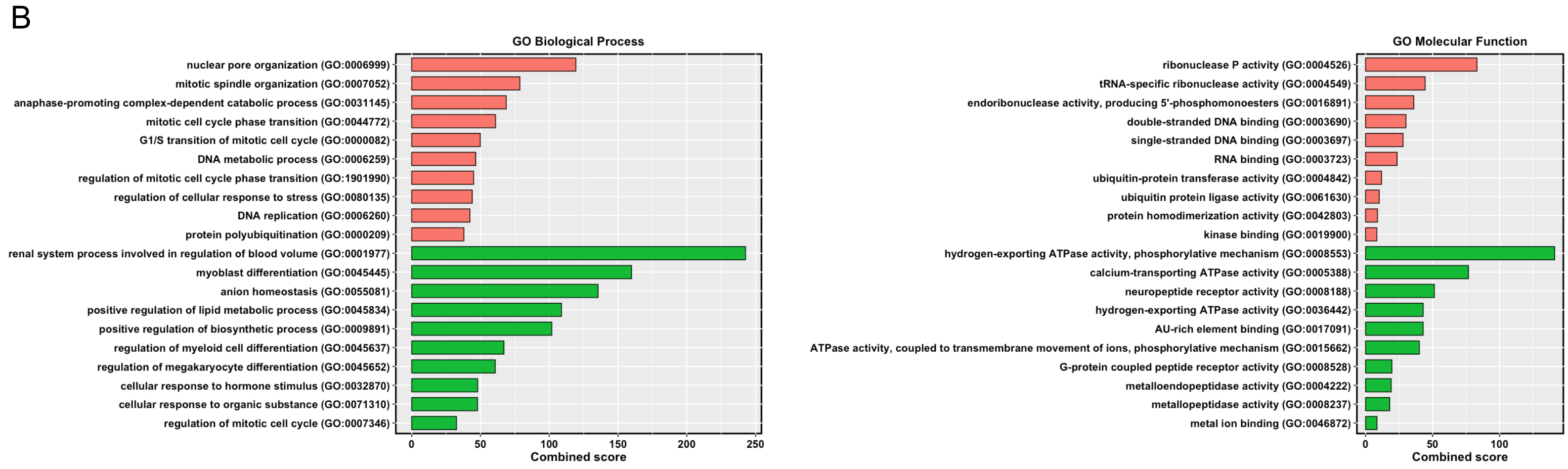

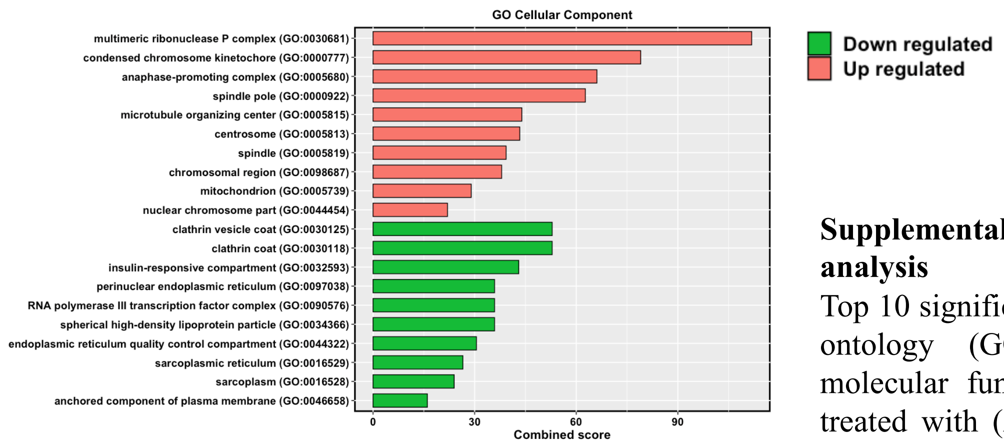

Supplemental Figure 2. Gene ontology enrichment analysis

Top 10 significantly upregulated and downregulated gene ontology (GO) categories in biological process, molecular function and cellular component in tumors treated with (A) durvalumab/oleclumab vs. durvalumab and (B) durvalumab/oleclumab vs. oleclumab.

Signature	Gene	Source
Inflammation	*PLA2G2A AGR2 AGR3 RAB27B GNB4 CLEC2B PLXNC1 DPYD MS4A7 APOE SPP1 LY96 CD109 MAFB DSE GPNMB CASP1 PBK TYMS APOC1 SLC2A3 TNFAIP6 BCAT1 DUSP4 MT1E LYZ RNF125 GZMA VNN1 RARRES1 CXCL13 CRIP1 MT2A DOCK8 MT1X RARRES3 SAMD9L PTPRC MT1H EVI2B CD52 HLA-DRA SAMSN1 CYTIP CD53 C1QB LCP2 HLA-DPB1 C1QA TRIM22 CD69 KYNU HLA-DMA HLA-DPA1 CCL5 SAMD9 CSF2RB IFI16 CCL8 BIRC3 SRGN BCL2A1 HCLS1 C1QC S100A8 RGS1 SLAMF8 ITGB2 TNFSF13B CD14 EVI2A ANXA1 LAPTM5 CXCR4 FCER1G NCF2 TYROBP CD163 MS4A4A CCL18 TFAP2A HOXC6 PMAIP1 HSPA6 PLA2G4A SRSF6 APOL6 GPR126 SEMG1 HSPA4L SLC7A11 EIF5A MOCOS ZNF165 GNLY HNRNPH1 FAS ZIC2 PSMB9 ANXA10 TFF2 IDO1 IFI6 CD74 CXCL10 FYB FSCN1 GBP1 IFIT3 SLAMF7 HLA-DMB BST2 BAG2 BTN3A3 IL1RN PLK2 CYBB CXCL9 TAGAP TRBC1 CCL4 MNDA CXCL11 GIMAP6 FAM26F IFI44 APOBEC3G MMP12 XAF1 RSAD2 OAS2 PLA2G7 IFIT2 GBP5 LCK TLR8 GBP4 IFI44L ISG15 MICB STAT1 CALB1 IGF2BP3 RPL22L1 TMPRSS3 TNFSF9 PIWIL1 LY6E CMPK2 AFAP1-AS1 AIM2 MS4A6A FCGR3B CLEC7A FPR3 FCGR1B*	Reference 47
Leukocyte infiltration	*ALOX5 ARHGAP30 BLNK CCR5 CD2 CD37 CD48 CD52 CD53 CD84 CD86 CECR1 COTL1 CTSS CXCR4 CYBB DOCK10 DOCK2 DOCK8 EVI2A EVI2B FCER1G FCGR1B FYB GLIPR1 GNG2 GPR137B GZMA GZMK HCLS1 HLA-DMB HLA-DPB1 HLA-DQB1 HLA-DRB1 IKZF1 IL10RA IL2RG IQGAP2 ITGA4 ITGAM ITGB2 KIAA1598 KLRK1 KYNU LAPTM5 LCK LCP1 LCP2 MPEG1 MREG NKG7 NPL PTPRC RBM47 RGS1 SLAMF7 SLAMF8 STK17B TLR8 TMEM176B TNFAIP8 WIPF1*	Reference 48
IFNγ response	*STAT1 ISG15 IFIT1 MX1 IFIT3 IFI35 IRF7 IFIT2 OAS2 TAP1 EIF2AK2 RSAD2 MX2 IRF1 OAS3 TNFSF10 IRF9 CXCL10 IFI44 BST2 XAF1 SP110 OASL PSMB8 IFI44L IFITM3 DDX60 LGALS3BP GBP4 IRF8 PSMB9 PML IFIH1 UBE2L6 IFI27 ADAR LY6E STAT2 CXCL9 IL10RA PLA2G4A TRIM21 USP18 PTGS2 EPSTI1 C1S DDX58 IL15 NLRC5 NMI IDO1 PSMB10 CXCL11 ITGB7 SAMHD1 HERC6 CMPK2 SAMD9L RTP4 PTPN2 PARP14 TNFAIP2 IFITM2 PLSCR1 SOCS1 CASP1 ICAM1 WARS PSME1 ISG20 IRF2 TRIM14 FCGR1A MARCH1 SOCS3 JAK2 HLA-DMA PARP12 TNFAIP6 TRIM26 VCAM1 CD274 CIITA NAMPT SELP GPR18 FPR1 PRIC285 PSME2 SERPING1 CCL5 RNF31 SOD2 TRIM25 LAP3 PSMA3 RNF213 PELI1 CFB CD86 TXNIP HLA-DQA1 GCH1 PNP CCL7 PTPN6 SPPL2A IL4R PNPT1 DHX58 BTG1 CASP8 IFI30 CCL2 FGL2 CASP7 SECTM1 IL15RA CD40 TRAFD1 HLA-DRB1 GBP6 LCP2 HLA-G MT2A RIPK1 KLRK1 UPP1 PSMB2 TDRD7 HIF1A EIF4E3 VAMP8 PFKP CD38 ZBP1 BANK1 TOR1B RBCK1 PDE4B MVP IL7 BPGM FTSJD2 AUTS2 B2M RIPK2 CD69 MYD88 PSMA2 PIM1 NOD1 CFH TAPBP SLC25A28 PTPN1 TNFAIP3 SSPN NUP93 MTHFD2 CDKN1A IRF4 NFKB1 BATF2 HLA-B LATS2 IRF5 SLAMF7 ISOC1 P2RY14 STAT3 NCOA3 HLA-A IL6 GZMA IFNAR2 CD74 RAPGEF6 CASP4 FAS OGFR ARL4A SRI LYSMD2 CSF2RB ST3GAL5 C1R CASP3 CMKLR1 NFKBIA METTL7B ST8SIA4 XCL1 IL2RB VAMP5 IL18BP ZNFX1 ARID5B APOL6 STAT4*	Reference 49
Type 1 IFN response	*EPSTI1 HERC5 IFI27 IFI44 IFI44L IFI6 IFIT1 IFIT3 ISG15 LAMP3 LY6E MX1 OAS1 OAS2 OAS3 PLSCR1 RSAD2 RTP4 SIGLEC1 SPATS2L USP18*	Reference 50
T cell cytotoxicity	*NKG7 CST7 PRF1 GZMA GZMB IFNG*	Unpublished

Supplemental Table 1. Genes in expression signatures
